# Corrigendum to “Bevacizumab, a vascular endothelial growth factor inhibitor, promotes orthodontic tooth movement in an experimental rat model” [Heliyon Volume 9, Issue 5, May 2023, Article e16217]

**DOI:** 10.1016/j.heliyon.2025.e43311

**Published:** 2025-04-17

**Authors:** Hatem Abuohashish, Abdulaziz Alamri, Suliman Shahin, Dalal Almazrou, Taleb Alkhamis, Omar Omar

**Affiliations:** a Department of Biomedical Dental Sciences, College of Dentistry, Imam Abdulrahman Bin Faisal University, Dammam, 31441, Saudi Arabia; b Department of Preventive Dental Sciences, College of Dentistry, Imam Abdulrahman Bin Faisal University, Dammam, 31441, Saudi Arabia; c Department of Environmental Health Research, Institute for Research and Medical Consultations, Imam Abdulrahman Bin Faisal University, Dammam, 31441, Saudi Arabia

Post-publication, an investigation conducted on behalf of the journal by Elsevier's Research Integrity & Publishing Ethics team identified a concern around the authenticity of the following figures/image panels: *Fig. 5 panel A, Bevacizumab-week1* appears to depict the same source image as another published source ‘Progress in Orthodontics, 2023 Oct 16; 24:33[ Page: 9], 10.1186/s40510-023-00486-z’, but are described as representing different experimental samples.

The authors were requested to provide comment on these concerns, as well as the original unprocessed image files to aid investigation. The authors have clarified that the image was unintentionally duplicated in the two articles due to human error during the preparation of the final layout, after the data analysis. The authors have provided the correct figure as below.

The original published Fig. 5 can be found below:

**Figure:**
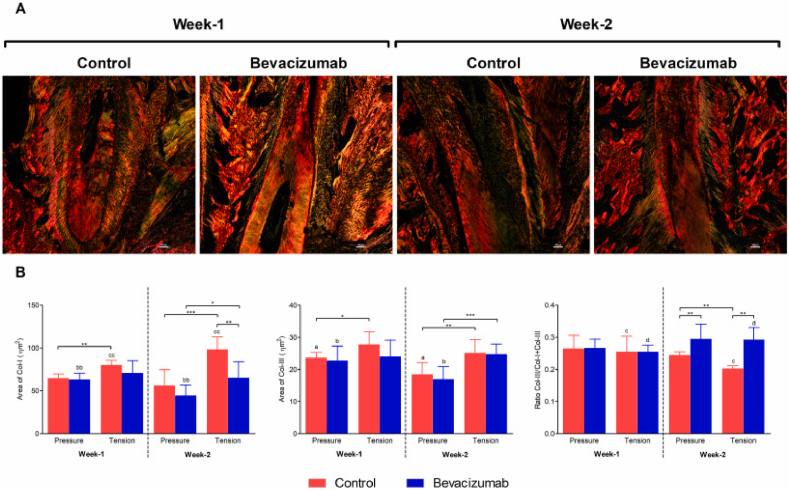

*Fig. 5 panel A, Bevacizumab-week1* has been corrected with the following:

**Figure:**
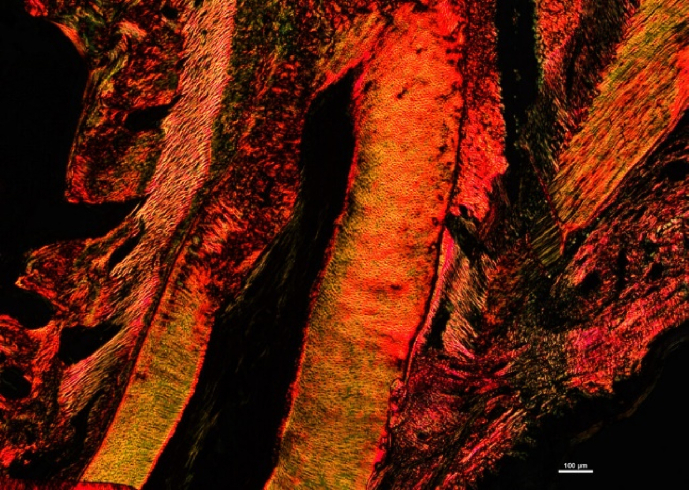

The correct version of Fig. 5 should be as below:

**Figure:**
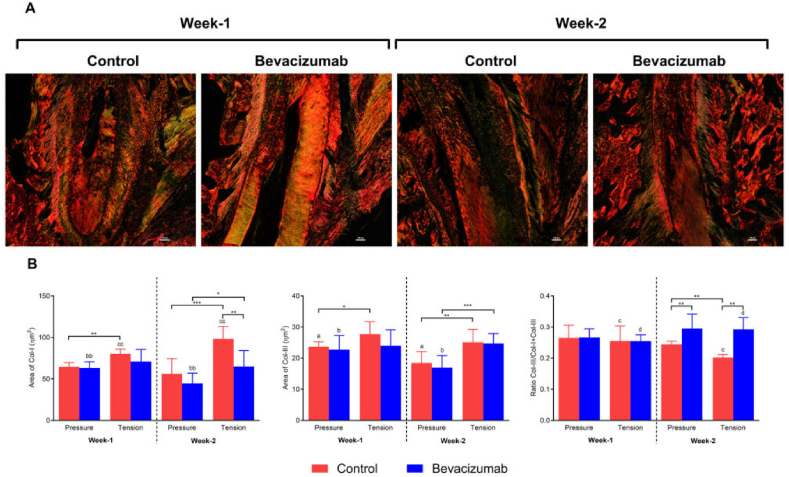

The *authors* apologize for the error.

## Declaration of competing interest

The authors declare that they have no known competing financial interests or personal relationships that could have appeared to influence the work reported in this paper.

